# Absorption Spectrum of Hydroperoxymethyl Thioformate: A Computational Chemistry Study

**DOI:** 10.3390/molecules30020338

**Published:** 2025-01-16

**Authors:** David Catalán-Fenollosa, Javier Carmona-García, Ana Borrego-Sánchez, Alfonso Saiz-Lopez, Daniel Roca-Sanjuán

**Affiliations:** 1 Instituto de Ciencia Molecular, Universitat de València, Apartado 22085, 46071 Valencia, Spain; dacafe2@alumni.uv.es (D.C.-F.); javier.carmona@uv.es (J.C.-G.); ana.maria.borrego@uv.es (A.B.-S.); 2 Centre for Computational Chemistry, School of Chemistry, University of Bristol, Cantocks Close, Bristol BS8 1TS, UK; 3 Department of Pharmacy and Pharmaceutical Technology and Parasitology, Universitat de València, Apartado 22085, 46071 Valencia, Spain; 4 Department of Atmospheric Chemistry and Climate, Institute of Physical Chemistry Blas Cabrera, Consejo Superior de Investigaciones Científicas, 28006 Madrid, Spain; a.saiz@csic.es

**Keywords:** hydroperoxymethyl thioformate, excited states, absorption spectrum, photolysis, Nuclear Ensemble Approach, CASPT2

## Abstract

Hydroperoxymethyl thioformate (or HPMTF) is a compound relevant to the chemistry of sulfur in the marine atmosphere. The chemical cycling of this molecule in the atmosphere is still uncertain due in part to the lack of accurate knowledge of its photolytic behavior. Only approximations based on the properties of its chromophores are used in previous studies. In this work, we calculated the absorption spectra of the molecule in gas and aqueous phases using the Nuclear Ensemble Approach (NEA) and the CASPT2 method. Furthermore, we used such information to obtain relative photolysis rates. We found that the chromophore approximation overestimates the photolysis rates in the gas phase by twice the value obtained with the NEA-CASPT2 protocol. Furthermore, for the aqueous phase, we predict a lower role of photolysis as compared to the gas phase.

## 1. Introduction

Sulfur chemistry has a significant impact on climate [1]. It presents negative aspects, such as the acid rain caused by anthropogenic emissions of sulfur dioxide [2], but also positive effects, for instance, the impact on the radiative balance of the Earth due to light dispersion and the generation of clouds caused by sulfates [3,4]. Due to its relevance, sulfur chemical transformations occurring in the Earth’s atmosphere have been deeply studied, and plenty of information is known nowadays. However, there are still aspects that remain unclear. This is the case of the chemistry of the remote marine atmosphere, where the role played by hydroperoxymethyl thioformate (HPMTF or HOOCH_2_ SCHO) is still not well established.

HPMTF was first proposed theoretically in 2015 by Wu et al. These authors stated that under conditions of a low concentration of the oxidants NO, HO_2_, and RO_2_, comparable to those in the remote marine atmosphere, the DMS molecule released by marine biota would yield HPMTF through an autoxidation process [5]. This proposal was experimentally confirmed by Berndt et al. in 2019 [6]. In 2020, Veres et al. identified the molecule in global-scale airborne observations and performed two simulations using the global chemical transport model CAM-Chem, with and without the autoxidation process [7]. The findings indicated a significant concentration of HPMTF in the marine atmosphere, especially at lower altitudes between 0 and 2 km over sea level, where the concentration of dimethyl sulfide (DMS) is higher. The data suggested that HPMTF is a major reservoir of marine sulfur, reducing the yield of SO_2_ and sulfates in the DMS emission zones. Due to the unknown reactivity of HPMTF, the simulations performed in the study only considered the reactivity with the hydroxyl radical, OH.

In 2021, Khan et al. published a paper on a new environmental model based on the 3D tropospheric chemistry and transport model STOCHEM-CRI [8,9]. Khan et al. introduced new sinks for HPMTF, in particular, photolysis, dry deposition, and wet deposition, for the first time. Overall, the model comprised the following processes for HPMTF: dry and wet deposition, two reactions with the OH radical, and two photolysis processes. The photolysis properties were predicted based on the reactivity of similar compounds known in atmospheric chemistry, and their photolytic rates were assumed to be equal to said reactivities. Thus, for the C-S bond cleavage, the information used was that of the C-C bond breaking of propanal in reaction 1. For O-O bond cleavage, the rates used were those of the analogous bond breaking of methyl peroxide in reaction 2:

(1) $$CH_{3}CH_{2}CHO+h\nu \to CH_{3}CH_{2}+HCO,$$

(2) $$CH_{3}OOH+h\nu \to CH_{3}O+OH.$$

Therefore, the absorption cross-sections of propanal and methyl peroxide and their photolysis rates were summed to estimate the value for HPMTF [8]. This assumption of approximating the spectroscopic and photochemical behavior of a species as the combination of those of the chromophore groups that compose it (referred to hereafter as chromophore approximation) is common in atmospheric models due to the difficulty of characterizing some atmospheric species and reactions. However, it may lead to erroneous qualitative and quantitative conclusions.

Khan et al. performed simulations introducing the new photolytic reactions on top of the previously established model. In the simulation using the recommended value by Jernigan et al. [10] for the reaction between HPMTF and OH, the outcomes show a loss of HPMTF of 46%. The sulfur reservoir behavior was then called into question.

Considering the scarce knowledge on the role of HPMTF in the sulfur atmospheric cycle and, especially, the approximations used in the literature about the response to light of the molecule, we carried out a computational study in this work aimed at the following:

- Studying the structural parameters of HPMTF in both the gas and aqueous phases;
- Calculating the absorption spectra of HPMTF in the gas phase and in the aqueous phase;
- Performing a critical analysis of the approximations used in the literature for the spectroscopic and photochemical properties of HPMTF;
- Estimating the photolytic behavior by calculating photolysis constants based on our computed spectra.

## 2. Results and Discussion

This section is divided into four main subsections. Firstly, we present and discuss the selection process of the computational method and the structural properties of HPMTF in the gas phase and aqueous phase. Secondly, excitation energies and oscillator strengths are analyzed for the ground state equilibrium structures of HPMTF in the isolated system and in water dielectric fields with and without explicit solvation molecules. Thirdly, the absorption electronic spectra are presented for the gas phase and aqueous solution. Finally, by using the computer spectra and information from the literature, photolysis rates are estimated. We also discuss the implications of the obtained findings in the field of marine sulfur chemistry and point to future directions of research to be carried out.

### 2.1. Geometrical Analyses

#### 2.1.1. Selection of Computational Method

The first step in the computational study of any molecule is the selection of the best method for describing the system, considering the computational cost and the quality of the results. We compared four methods: Hartree–Fock (HF), Density Functional Theory (DFT), Møller–Plesset second-order perturbation theory (MP2), and Coupled Cluster Singles and Doubles (CCSD).

**Figure 1:**
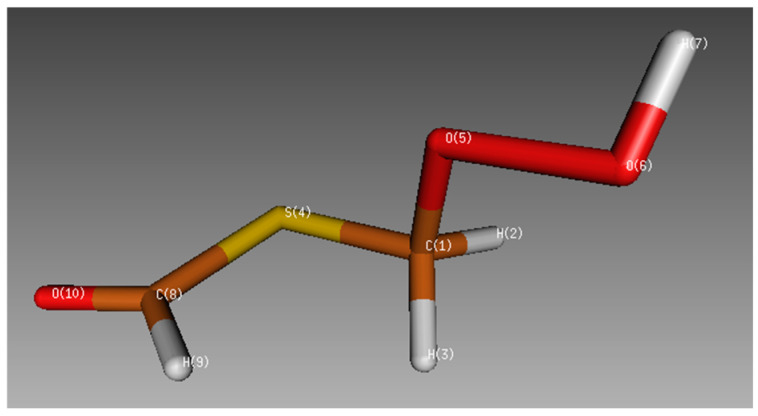
Optimized HPMTF structure with the DFT B3LYP method and the 6-311++G(d,p) basis set. Atom labeling and numbering are shown.

After finding the same equilibrium structure by geometry optimization of the HPMTF molecule using each method, we compared the results. Table 1 presents all the bond distances and the most relevant angles (see labeling and numbering in Figure 1). As there are no previous computational or experimental studies available on the structural characterization of this molecule, the highest-level method (CCSD) is chosen as the reference method [11].

The structural parameters of Table 1 do not show drastic differences for any method. However, a significant difference can be observed in the bond distances and bond angles including oxygen atoms for the Hartree–Fock (HF) method. This discrepancy is related to the lack of electron correlation in this method. Therefore, the HF method is excluded from the upcoming analyses. The remaining methods present similar structural values, so we analyzed the frequency values of the vibrational modes to compare their performance (see Table 2).

The frequency values obtained with the three methods do not deviate significantly. Considering the CCSD method as the reference method, as mentioned before, the MP2 method has an average relative error of −0.5%, and the DFT method has an average relative error of −4.4%. Given the fact that the DFT method is the least computationally expensive method, and it produces results comparable to the reference method CCSD, we selected the DFT B3LYP method for all subsequent calculations, since the calculations in the aqueous phase are much more computationally expensive.

#### 2.1.2. Conformational Study of HPMTF

Once the electronic structure method is selected, we explore the conformational space of HPMTF in the gas phase. By starting from the equilibrium structure used in the previous section and performing a metadynamics simulation with the GFN-FF force field in the CREST software (version 3.0.1), we obtained five additional conformers, each having two possible energetically degenerate rotamers (a total of 12 conformers and rotamers). The rotamers differ in the orientation of the peroxide moiety with respect to the plane defined by the atoms S4, C8, H9, and O10. For example, the structure of HPMTF in Figure 1, Structure 12, gives rise to the rotamer Structure 11 by applying a 180-degree torsion of the O5-C1-S4-C8 dihedral angle.

**Figure 2:**
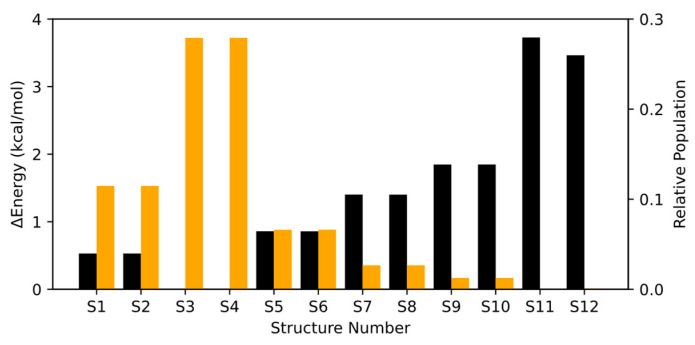
Relative energies (black) and Boltzmann populations (orange) for all conformers and rotamers in the gas phase.

The structures obtained from the metadynamics simulation were optimized using the DFT B3LYP method (see Supplementary Information for the cartesian coordinates of all conformers and rotamers), along with the initial structure and its corresponding rotamer. The twelve conformers and rotamers are represented in Figure 2, and the exact values for the energies and populations are given in Table S1. As seen in Figure 2, after optimizing the structures at the DFT levels, the energies of each pair of rotamers maintain the same energy. It is worth noting that even though the structure employed for the method benchmarking in the previous section (Structure 12) is not the most stable, the same conclusions will be obtained by selecting any other isomer.

#### 2.1.3. Water Clusters and Aqueous Solution

The absorption spectrum in the aqueous phase of HPMTF requires the optimization of the molecule in an aqueous environment. Each structure was calculated by introducing a Polarizable Continuum Model (PCM) field to its gas phase optimized geometry. Then, we optimized the obtained structures considering the PCM field and explicit water molecules to represent the first solvation shell. These calculations were performed with the DFT B3LYP method and included Grimme’s D3 dispersion correction. The number of water molecules ranged from three up to six, considering possible hydrogen bond interactions on the oxygen of the carbonyl group, on the sulfur atom, on the oxygen atoms of the peroxide group, and on the hydrogen bonded to the peroxide. The models with three and four water molecules showed stable hydrogen bond interactions. On the contrary, when adding extra water molecules, they do not maintain the solute–solvent interaction when the geometry is optimized. The atoms that did not maintain the interaction with the solvent were the sulfur atom and one of the oxygen atoms of the peroxide group. Therefore, we considered the cluster with four explicit water molecules as the solvated state of HPMTF.

Analyzing the structural parameters, we observed that, on average, the solvation of the conformers and rotamers elongated the bonds O6-H7 and C8-O10 by 2.09% and 1.46%, respectively, while it shortened the bond S4-C8 by 1.65%. These variations are analogous to other molecules with a carbonyl group, such as acrolein.

**Figure 3:**
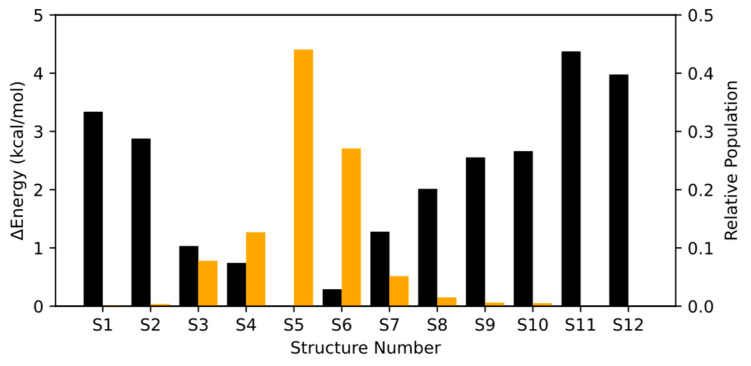
Relative energies (black) and Boltzmann populations (orange) for all structures in the aqueous phase.

The relative energy and Boltzmann population of all conformers and rotamers in the aqueous phase is represented in Figure 3 (see Table S2 for exact values). There are two aspects worth noting. First, the energies of the pair of rotamers are not as degenerate as in the gas phase. This is caused by the increased degrees of freedom in the calculations due to the solvent molecules, which increases the number of equilibrium states in the potential energy surface. Second, there is a change in the Boltzmann distribution in comparison to gas phase. This is caused by the fact that Structures 5 and 6 have a water molecule with two hydrogen bonds, one with the oxygen of the carbonyl group and one with the oxygen of the peroxide group, which greatly stabilizes this conformer with respect to the other conformers and rotamers. The optimized structures for all rotamers are given in the Supplemental Information in xyz format.

### 2.2. Excitation Properties: Nature, Energies, and Oscillator Strengths

#### 2.2.1. Isolated Molecule

The nature of the excited states, vertical excitation energies, and oscillator strengths were computed herein for the ground state equilibrium structures of the molecule with the Complete Active Space Self Consistent Field (CASSCF) method and Complete Active Space Perturbation Theory up to second order (CASPT2) method. These methodologies require two important aspects to be set up: the active space and the number of electronic states (or roots) included. This depends heavily on the goal of the study. Here, we focus on the vertical excitations that can be produced by the predominant solar radiation arriving at the troposphere, the UV-Vis region (280–700 nm or 1.8–4.4 eV).

**Figure 4:**
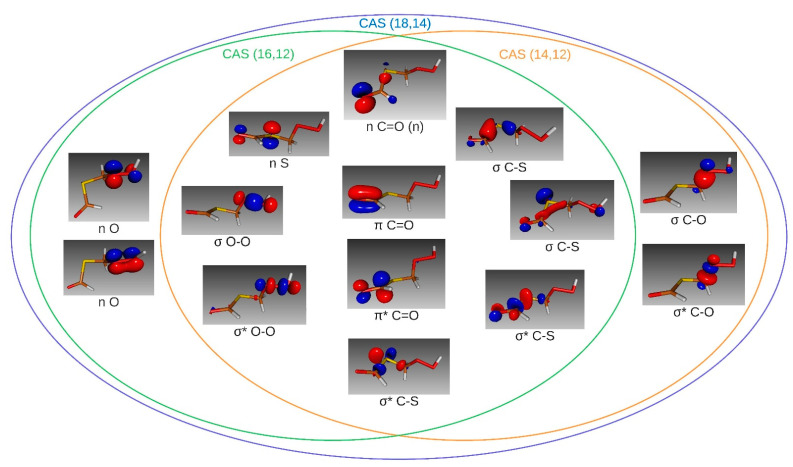
Venn diagram for the molecular orbitals of the three active spaces: (18,14), (16,12), and (14,12). The nomenclature used for the molecular orbitals is given below each orbital. The lone pair of the C=O group is also referred to as simply *n* due to its common appearance in this work.

For the mentioned range of energies, the corresponding electronic transitions especially involve the π, π*, and lone pair (*n*) orbitals. Furthermore, the σ and σ* orbitals of the weakest bonds may also have non-negligible contributions to these transitions. The first active space selected included all orbitals that may fit the desired characteristics: the lone pairs of the atoms S4, O5, O6, and O10, the π and π* orbitals of the carbonyl group, and the σ and σ* orbitals of the C8-S4, S4-C1, C1-O5, and O5-O6 bonds. This corresponds to a complete active space of 18 electrons distributed in 14 orbitals, that is, CAS (18,14), which is shown in Figure 4. Two additional active spaces were computed to analyze the effect of removing orbitals lowly contributing to the vertical excitation energies and oscillator strengths. These active spaces were derived from the “inactivation” of the lone pair orbitals of the peroxide group, CAS (14,12), or the σ and σ* orbitals of the C1-O5 bond, CAS (16,12).

Comparisons of the performance of the distinct active spaces were performed with Structure 12, although the same conclusions will be derived for the other conformers and rotamers. All three active spaces included eight electronic states. The vertical excitation energies and oscillator strengths of each calculation are presented in Table 3. Focusing on the UV-Vis region, the most relevant transition for HPMTF in the troposphere is the excitation from the electronic ground state, S_0_, to the first excited state, S_1_. However, the transitions from the ground state, S_0_, to the second excited state, S_2_, and third excited state, S_3_, may have some interest in structures that have been slightly distorted due to the vibrations of the molecule.

By analyzing the transitions shown in Table 3, we can distinguish that the S_0_ to S_2_ transition that appears in the active spaces (18,14) and (16,12) is not described in the space (14,12). Moreover, the excitation energy of the transition S_0_ to S_1_ differs slightly compared to the other two active spaces. Therefore, we rejected CAS (14,12) as an adequate space. The remaining active spaces show good agreement in the transitions of interest mentioned above, both in excitation energies and oscillator strengths. These results imply that the lone pairs of the peroxide group are significant, while the σ and σ* orbitals of the bond C1-O5 are irrelevant to the electronic behavior of HPMTF. Since the computational cost of the CAS (16,12) calculation is less than 5% with respect to the CAS (18,14) calculation, we performed all the following electronic structure calculations with CAS (16,12).

Once the active space was selected, we studied the effect of the number of electronic states included in the calculations. Generally, calculations should include several roots more than those of interest to capture possible differential correlation effects between CASSCF and CASPT2. Therefore, we analyzed the effect of including four, six, and eight roots in the four electronic states mentioned above. The results of these calculations are presented in Table 4. The vertical excitation energies and the oscillator strengths of all three calculations do not deviate significantly among the excited states of interest. Hence, we chose to include eight electronic states in all the following electronic calculations due to its reduced computational cost.

The analysis of the nature of the electronic excitations was focused on the first excited state since it is the predominant transition in the UV-Vis region. All conformers and rotamers were studied for this analysis.

The electronic excitation from the electronic ground state, S_0_, to the first excited state, S_1_, is characterized mainly by the excitation of an electron from the lone pair of the oxygen atom of the carbonyl group to the π* orbital of the same carbonyl group, which accounts for an average of 84.7% across all structures. There are other excitations that contribute to the first excited state, but their contributions have values around 2% or even lower and can be considered as negligible. The lone pair to π* transition, also referred to as *n* to π*, is usual in systems with carbonyl groups, such as acrolein [12], and it is highly characteristic. It is lower in energy than the transition from π to π* due to the higher energy of the lone pair compared to that of the π orbital. Moreover, it is known as a dark state because of the selection rules and the symmetry of both MOs, which leads to low values of the oscillator strength. The vertical excitation energies and oscillator strengths of each pair of rotamers are almost identical, which is reasonable when considering their degenerate nature (shown in Figure 2).

#### 2.2.2. Solvatochromic Shifts

The electronic transitions calculated in the aqueous phase are affected by two factors with respect to the gas phase calculations, the distinct structural parameters of the molecule, and the interactions of the solvent and solute. To study the effects of each factor, we performed electronic structure calculations for Structure 12. The effects of these two factors can be deduced from the results compiled in Table 5.

Two opposite shifts can be observed in Table 5. Focusing on the first excited state, the “No Field” calculation presents a red shift caused by the elongation of the C8-O10 bond mentioned above since the elongation causes a weakening of the bond. The inclusion of the point charge field (“Field” values) causes a blue shift because the charge field stabilizes the lone pair orbital with respect to the π* orbital. The net effect of the solvation of HPMTF for the first excited state is negligible, which may indicate that the absorption spectra for the gas phase and aqueous phase are similar. As a comparison, the solvation of acrolein presents a net blue shift for the *n* to π* transition. Regarding the oscillator strength values, there are no significant deviations across the calculations from the gas phase to the aqueous phase.

### 2.3. Absorption Electronic Spectra

#### 2.3.1. Cross-Sections in the Gas Phase

The gas phase spectrum was generated following the Nuclear Ensemble Approach (NEA), where the vertical excitation energies and oscillator strengths are calculated for a sample set of geometries and a phenomenological broadening is applied to obtain the spectrum. The gas phase spectrum of HPMTF, assuming a tropospheric temperature of 300 K, is calculated by performing a weighted average of the absorption spectrum of each conformer or rotamer depending on their relative Boltzmann populations. The absorption spectrum of each isomer was calculated considering a nuclear ensemble of 200 structures and a broadening of 0.2 eV (the absorption cross-sections of each conformer are given in Table S3). According to Figure 2, the presence of the isomers Structures 11 and 12 in the troposphere is negligible, so its absorption spectrum can be omitted.

**Figure 5:**
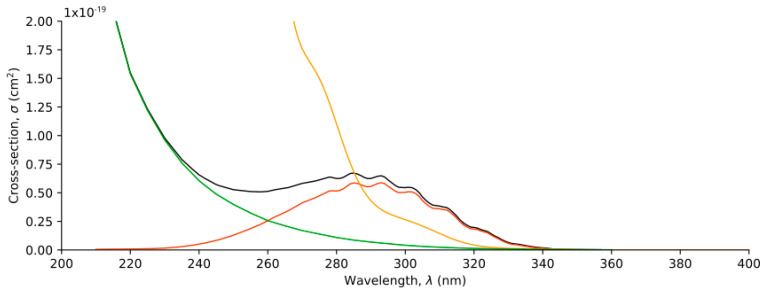
Gas phase absorption spectra for propanal (red line), methyl peroxide (green line), the sum of the two spectra (black line), and the HPMTF spectrum computed in this work (yellow line).

The calculated gas phase spectrum of HPMTF is represented in Figure 5, along with three additional spectra. Two of them are the absorption spectra of propanal and methyl peroxide [13], which were used to estimate the photolysis of HPMTF in Ref. [8]. The last spectrum is the sum of the absorption cross-sections of both compounds, which is the expected spectrum of HPMTF according to the chromophore approximation.

As seen in Figure 5, the reference spectrum of HPMTF obtained by the chromophore approximation does not correspond to the calculated gas phase spectrum. The calculated spectrum has higher cross-section values in the high-energy region (λ < 280 nm), while the absorption cross-section values are significantly lower in the UV-Vis region (λ > 280 nm). These deviations are caused by the different molecular environments of each compound, especially for the chromophore groups and the interactions among them.

#### 2.3.2. Cross-Sections in Aqueous Solution

The aqueous phase absorption spectrum of HPMTF was calculated following an analogous procedure. Computational details related to the description of the aqueous phase are discussed in the methodology section. In this case, we calculated the absorption spectra of Structures 3–8, since the other conformers and rotamers had negligible relative Boltzmann populations according to Figure 3.

**Figure 6:**
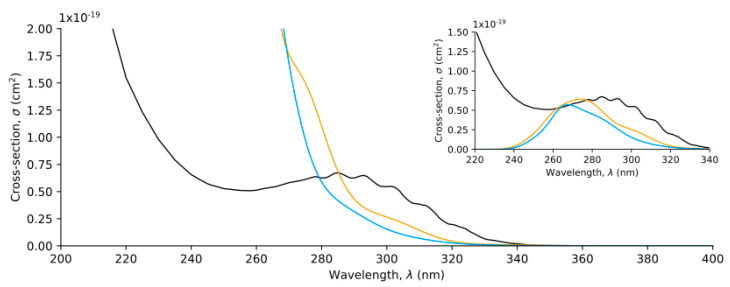
Absorption spectra for the chromophore approximation, black line; the calculated gas phase HPMTF, yellow line; and the aqueous phase HPMTF, blue line. The graphic at the corner represents the absorption spectra corresponding to the first electronic excitation (S_0_ to S_1_).

The aqueous phase absorption spectrum of HPMTF is represented in Figure 6, along with the gas phase spectrum and the chromophore approximation spectrum. The cross-section values of each isomer are given in Table S4. The absorption spectrum of each phase corresponding exclusively to the first excited state is given in Figure 6 as an inset. The first excited state does not suffer any major solvatochromic shifts, as already anticipated in the analysis above. However, the cross-section values of the aqueous phase decrease slightly with respect to the gas phase values.

### 2.4. Estimated Photolysis, Atmospheric Implications, and Future Perspectives

The photolysis constants were estimated for the gas and aqueous phases of HPMTF and the chromophore approximation, so the values obtained could be comparable to the previous bibliography. The photolysis rates and lifetimes are given in Table 6. Note that photolysis rates in this work are an upper limit since the quantum yield of photolysis is approximated to 1. Non-adiabatic molecular dynamics simulations must be conducted in the future to clearly determine these values. Nevertheless, the current numbers already allow for an analysis of the quality of the chromophore approximation and the effect of the aqueous environment on the excited state chemistry of HPMTF.

As seen in Table 6, the gas phase photolysis calculated in this work is significantly lower than the photolysis rate of the chromophore approximation, 31.5% of its value. This indicates that applying said approximation to HPMTF leads to the overestimation of the photolytic loss in the troposphere. It is worth noting that we observed some electronic excitations that would not be possible while using the chromophore approximation. This transition was the excitation of an electron of the lone pair of the carbonyl group to the σ* orbital of the peroxide bond and was especially present in Structure 12, around 37%. This transition may suggest the possibility of a new photolytic pathway apart from reactions 1 and 2. As pointed out above, non-adiabatic molecular dynamics will allow us to decipher the efficiency of this new chemistry.

Regarding the aqueous phase conditions, present in the marine troposphere, our findings seem to point to a higher role of HPMTF as a reservoir of sulfur, extending the lifetime by almost a factor of two with respect to the gas phase. Veres et al. pointed out the rapid consumption of HPMTF in clouds, even with respect to its precursor DMS [7]. A recent work by Rao et al. studied and proposed a new oxidation process for thiols and thioethers in sea spray aerosol at the air–water interface [14]. In this work, some species of thioether spontaneously oxidized into sulfoxide and sulfone products. We suggest that HPMTF may suffer this oxidation process to yield the analogous sulfoxide and sulfone compounds, although the mechanism will have to be studied together with the photolysis yields to clearly understand the general behavior in water droplets.

## 3. Materials and Methods

The computational methods selected for the optimization of the HPMTF structures were compared in the gas phase. The methods used were the HF method, the post-HF MP2 and CCSD methods [15,16,17], and the DFT electron density method with the B3LYP functional [18,19,20,21]. All methods used the Pople basis set of 6-311++G(d,p) quality [22,23,24,25,26].

The conformational study of HPMTF in the gas phase consisted of a metadynamics simulation using the GFN-FF method with the software Conformer-Rotamer Ensemble Sampling Tool (CREST, version 3.0.1) [27,28,29,30]. The structures obtained were optimized with the selected computational method, B3LYP. The rotamers of HPMTF in the aqueous phase were optimized using the PCM model and several explicit water molecules for the first solvation shell [31,32,33,34]. The method used was B3LYP and the D3 dispersion correction of Grimme [35]. All optimizations were performed using the software package Gaussian (version G16, revision C.01) [36].

The electronic spectra were calculated considering the Nuclear Ensemble Approach [37]. For the gas phase absorption spectra calculations, we used the standard procedure of the MultiSpec program (version 1.0) [38]. The nuclear ensembles were generated using the Wigner distribution and the Newton-X software package (version 2.0) [39,40]. All ensembles had 200 structures. The vibrational modes of the aqueous phase calculations under 100 cm^−1^ were set as ‘frozen’ while performing the Wigner distribution, which means that they were not taken into consideration for the ensemble. The reason was that the anharmonic nature of these vibrations (caused by the explicit water molecules) led to structures with broken bonds, which did not represent the molecule adequately.

To study the aqueous phase conditions, the macromolecular system was generated by molecular dynamics (MD) simulations using the AMBER 22 software [41,42], following the standard procedure established in the MultiSpec software (version 1.0) [38]. The initial configuration of the system was built by introducing the structure of HPMTF optimized with PCM and explicit water molecules in a simulation box formed by 3047 water molecules whose initial dimensions were 47.686 × 51.636 × 48.997 Ångström. Once this initial configuration was generated, the HPMTF molecule was ‘frozen’ so that the coordinates of its constituent atoms remained fixed throughout the MD simulation. This was followed by a minimization stage, in which the coordinates of the waters forming the system were optimized, and a heating stage, in which a simulation was carried out by applying periodic boundary conditions and keeping the number of particles (N), the temperature (T), and the volume (V) of the system constant, resulting in a canonical (NVT) simulation. During the heating stage, the temperature of the system progressively increased from 100 K to 298 K for 20 picoseconds. Once the target temperature was reached, an equilibrium step was performed using a 1 nanosecond simulation, in which periodic boundary conditions were imposed, and the temperature at 298 K and the pressure (P) at 1.013 bar were kept constant, as well as N, which is known as an isothermal–isobaric (NPT) simulation. Starting at 400 picoseconds into the simulation, when the system reached a steady state, one frame was extracted every 25 picoseconds for a total of 25 frames. Using the procedure explained previously, 200 new initial configurations were generated, replacing the MD solute geometry in the 25 frames with the 200 Wigner geometries of the solute (8 structures per frame) adjusted by the tool program of MultiSpec. For each of the initial configurations generated, a 5-picosecond NVT simulation was performed, with the HPMTF coordinates fixed, to allow the water molecules to adapt to the new structure. Finally, the last frame of each simulation was extracted to calculate the spectrum. The effect of the water molecules in the systems was considered in the electronic states calculation as an external electric field by point charges, which corresponded to the coordinates and partial charges of the oxygen and hydrogen atoms in the water molecules.

Regarding the force field, the General Amber Force Field (GAFF) parameters were used for the HPMTF [43], and for the water molecules, the TIP3P water model was used along with the SHAKE algorithm to restrict the bond lengths where hydrogen was involved (not applicable to the HPMTF bonds) [44,45]. The HPMTF partial charges were calculated with the Gaussian program at the same level of theory as the aqueous optimizations and using the Merz–Singh–Kollman (MK) method [46,47]. Temperature and pressure throughout the simulations were controlled with the Langevin thermostat and the Berendsen barostat, respectively. The integration step during all stages was 1 femtosecond. The cutoff radius considered for short-range interactions was 10.0 Ångström, while long-range interactions were evaluated with a modification of the Ewald algorithm called PME (Particle Mesh Ewald) [48,49].

The electronic spectroscopy calculations were performed using the methods SA-CASSCF and MS-CASPT2 [50,51]. The basis used was the atomic natural orbitals with triple-zeta (ζ) and polarization (ANO-L-VTZP) [52,53]. Several active spaces and averaged electronic states were considered to find the most adequate space (see discussions in the work). All electronic states considered were singlets. The MS-CASPT2 method included the IPEA shift of 0.25 a.u. [54], as in related previous studies on similar molecules, and an imaginary shift of 0.2 a.u. to minimize the effect of intruder states [55,56,57]. The spectroscopic calculations were performed with the OpenMolcas software package (version 18.09) [58,59,60]. The absorption spectra were obtained by using Gaussian functions with width values of 0.2 eV at half maximum. Finally, the absorption spectrum for each phase was calculated by weighted averages depending on the relative Boltzmann population distribution of each rotamer considered in each phase.

The photolysis constants (*J*) were calculated according to Equation (3):

(3) $$J=\int \phi \left(\lambda \right)\sigma \left(\lambda \right)I\left(\lambda ,\theta \right)d\lambda ,$$

where $\phi \left(\lambda \right),\;\sigma \left(\lambda \right)$, and $I\left(\lambda ,\theta \right)$ are the quantum yield, the characteristic absorption cross-section of the molecule, and the actinide flux of incident radiation, respectively. The actinide flux of radiation depends on several factors (zenith angle, albedo, and height) and was calculated through an online application provided by the National Center for Atmospheric Research [61]. The values considered for the parameters were as follows: maximum quantum yield, equal to one; zenith angle of highest incidence, equal to zero; typical ocean albedo, equal to 0.1; sea surface height, equal to 0 km; and standard values for the calculation of I of solar radiation.

## 4. Conclusions

Hydroperoxymethyl thioformate (HPMTF) is a sulfur species discovered in the last decade and has been suggested to have a crucial role in sulfur cycling in the marine atmosphere. The atmospheric fate of HPMTF is currently uncertain, and some pathways have been proposed, such as bimolecular reaction with hydroxyl radicals, dry and wet deposition, and photolysis. The latest atmospheric study on HPMTF has suggested photolytic loss as the main sink, estimating the photolytic properties as a combination of those of simpler molecules with the same chromophore groups as HPMTF, in particular, propanal and methyl peroxide.

In this work, the absorption spectra of HPMTF have been computationally predicted by simulating both the gas and aqueous phases. The chromophoric approximation used in the literature is not found to give unrealistic data. However, lower absorption cross-sections are obtained herein for the real molecule of HPMTF in the atmospheric window (>290 nm). The calculated photolysis rate in the gas phase is predicted to be half the value obtained with the chromophoric approximation, and it is even lower for the aqueous phase. Further research is still needed to accurately predict the photolytic fragmentation of HMPTF and quantify the photolysis yields more accurately.

## Figures and Tables

**Table 1:** Structural parameters optimized for HPMTF using several computational methods (HF, DFT, MP2, and CCSD).

		HF	DFT	MP2	CCSD
Bond Distances (Å)	O10-C8 *	1.173	1.195	1.205	1.198
	C8-H9	1.093	1.106	1.107	1.107
	C8-S4	1.781	1.801	1.779	1.787
	S4-C1	1.799	1.812	1.791	1.800
	C1-H2	1.083	1.093	1.093	1.095
	C1-H3	1.083	1.094	1.095	1.095
	C1-O5	1.398	1.419	1.417	1.418
	O5-O6	1.385	1.466	1.460	1.446
	O6-H7	0.944	0.968	0.966	0.964
Bond Angles (°)	O10-C8-S4	122.4	123.0	123.5	123.1
	C8-S4-C1	99.9	99.2	97.5	97.8
	S4-C1-O5	108.4	106.7	106.3	106.9
	C1-O5-O6	107.0	105.5	104.7	105.2
	O5-O6-H7	102.7	99.9	99.0	100.0
Dihedral Angles (°)	O10-C8-S4-C1	178.7	178.6	176.8	177.0
	C8-S4-C1-O5	−73.9	−81.6	−77.1	−75.9
	S4-C1-O5-O6	178.2	−179.3	178.5	178.4
	C1-O5-O6-H7	126.7	130.6	130.0	127.8

* Atomic labeling and numbering corresponding to Figure 1.

**Table 2:** Frequency values (in cm^−1^) of the normal vibrational modes of HPMTF calculated using the methods DFT B3LYP, MP2, and CCSD.

Vibrational Mode	DFT	MP2	CCSD	Vibrational Mode	DFT	MP2	CCSD
1	57.3	63.9	64.4	13	963.0	989.1	993.0
2	103.3	116.9	113.2	14	1020.7	1069.2	1087.1
3	167.6	170.1	161.2	15	1219.9	1247.8	1256.0
4	188.1	216.4	223.0	16	1313.3	1336.6	1362.4
5	228.9	249.5	247.9	17	1356.8	1380.8	1407.5
6	273.9	288.0	291.9	18	1373.0	1406.6	1419.1
7	369.0	388.5	390.6	19	1492.5	1505.4	1515.2
8	475.0	495.8	497.4	20	1788.6	1746.6	1806.1
9	741.0	788.2	785.3	21	2929.9	2990.9	2990.7
10	772.4	823.4	820.5	22	3033.8	3090.1	3078.5
11	879.8	877.5	926.5	23	3092.2	3156.8	3135.2
12	924.3	934.3	940.2	24	3778.9	3830.8	3854.7

**Table 3:** CASPT2 vertical excitation energies (ΔE), in eV, and oscillator strengths for the CAS (18,14), (16,12), and (14,12) approaches including 8 electronic states performed for Structure 12.

Electronic State	CAS (18,14)		CAS (16,12)		CAS (14,12)	
	ΔE (eV)	Osc. Str.	ΔE (eV)	Osc. Str.	ΔE (eV)	Osc. Str.
S_0_	0.0000		0.0000		0.0000	
S_1_	4.5869	1.33 × 10^−4^	4.5821	1.26 × 10^−4^	4.6182	1.40 × 10^−4^
S_2_	5.5525	5.95 × 10^−3^	5.5657	3.53 × 10^−3^	5.7950	6.33 × 10^−2^
S_3_	5.7562	8.73 × 10^−2^	5.7931	8.12 × 10^−2^	6.1512	1.13 × 10^−3^
S_4_	6.1793	7.68 × 10^−4^	6.1035	8.01 × 10^−4^	6.9595	9.07 × 10^−2^
S_5_	6.9185	8.83 × 10^−2^	6.9377	1.12 × 10^−1^	8.0963	1.14 × 10^−2^
S_6_	7.5116	2.22 × 10^−4^	7.5587	1.78 × 10^−4^	9.2233	7.90 × 10^−2^
S_7_	7.8580	1.00 × 10^−2^	8.0324	1.55 × 10^−2^	10.3142	6.76 × 10^−4^

**Table 4:** CASPT2 vertical excitation energies (ΔE), in eV, and oscillator strengths for the CAS (16,12) approach including 8, 10, and 12 electronic states, performed for Structure 12.

Electronic State	8 Roots		10 Roots		12 Roots	
	ΔE (eV)	Osc. Str.	ΔE (eV)	Osc. Str.	ΔE (eV)	Osc. Str.
S_0_	0.0000		0.0000		0.0000	
S_1_	4.5821	1.26 × 10^−4^	4.5798	1.22 × 10^−4^	4.6082	9.65 × 10^−5^
S_2_	5.5657	3.53 × 10^−3^	5.5101	4.51 × 10^−3^	5.4983	5.02 × 10^−3^
S_3_	5.7931	8.12 × 10^−2^	5.7570	8.90 × 10^−2^	5.7677	7.41 × 10^−2^
S_4_	6.1035	8.01 × 10^−4^	6.2168	7.34 × 10^−4^	6.2052	1.15 × 10^−3^
S_5_	6.9377	1.12 × 10^−1^	6.9639	1.01 × 10^−1^	6.9461	9.87 × 10^−2^
S_6_	7.5587	1.78 × 10^−4^	7.6567	3.20 × 10^−3^	7.4712	1.90 × 10^−3^
S_7_	8.0324	1.55 × 10^−2^	7.7128	1.48 × 10^−3^	7.7658	2.69 × 10^−4^
S_8_			8.1640	1.41 × 10^−2^	8.0708	1.29 × 10^−2^
S_9_			8.7098	2.06 × 10^−3^	8.1414	1.37 × 10^−2^
S_10_					8.7480	1.48 × 10^−3^
S_11_					9.3123	7.55 × 10^−2^

**Table 5:** CASPT2 vertical excitation energies (ΔE), in eV, and oscillator strengths for the CAS (16,12) approach including 8 electronic states and performed for Structure 12 in the water continuum model and explicit solvation. The “No Field” and “Field” calculations were performed without and with the external field of charges corresponding to the water molecules, respectively.

Electronic State	No Field		Field	
	ΔE (eV)	Osc. Str.	ΔE (eV)	Osc. Str.
S_0_	0.0000		0.0000	
S_1_	4.4374	6.17 × 10^−5^	4.5649	2.54 × 10^−4^
S_2_	5.5770	9.64 × 10^−2^	5.6615	1.15 × 10^−1^
S_3_	5.9234	1.14 × 10^−3^	5.8150	4.02 × 10^−4^
S_4_	5.9779	1.49 × 10^−3^	6.4215	1.68 × 10^−3^
S_5_	6.8171	7.66 × 10^−2^	7.1528	7.61 × 10^−2^
S_6_	7.3893	5.79 × 10^−4^	7.3539	1.86 × 10^−3^
S_7_	8.0283	1.52 × 10^−2^	7.7733	1.41 × 10^−2^

**Table 6:** Photolysis rate constants ($s^{-1}$) and mean lifetimes (hours) calculated for each absorption spectrum: chromophore approximation, gas phase, and aqueous phase.

	Photolysis Rate $\left(s^{-1}\right)$	Lifetime (Hours)
Chromophore Approximation	6.14 × 10^−5^	4.52
Gas Phase	1.93 × 10^−5^	14.37
Aqueous Phase	1.02 × 10^−5^	27.18

## Data Availability

The original contributions presented in the study are included in the article, and further inquiries can be directed to the corresponding author.

## Supplementary Materials

The following supporting information can be downloaded at https://www.mdpi.com/article/10.3390/molecules30020338/s1: Cartesian coordinates of structures, Spreadsheet: Energies and Boltzmann populations of structures, Spreadsheet: Absorption cross-sections in the gas and aqueous phase. Table S1: Energies & Distributions of gas phase HPMTF (conformers and rotamers). Table S2: Energies & Distributions of aqueous phase HPMTF (conformers and rotamers). Table S3: Gas phase Absorption Spectrum HPMTF. Table S4: Aqueous phase Absorption Spectrum HPMTF.
